# Synthesis and Characterization of Maghemite Nanoparticles Functionalized with Poly(Sodium 4-Styrene Sulfonate) Saloplastic and Its Acute Ecotoxicological Impact on the Cladoceran *Daphnia magna*

**DOI:** 10.3390/polym16111581

**Published:** 2024-06-03

**Authors:** Juan A. Ramos-Guivar, Renzo Rueda-Vellasmin, Erich V. Manrique-Castillo, F. Mendoza-Villa, Noemi-Raquel Checca-Huaman, Edson C. Passamani

**Affiliations:** 1 Grupo de Investigación de Nanotecnología Aplicada para Biorremediación Ambiental, Energía, Biomedicina y Agricultura (NANOTECH), Facultad de Ciencias Físicas, Universidad Nacional Mayor de San Marcos, Av. Venezuela Cdra 34 S/N, Ciudad Universitaria, Lima 15081, Peru; juan.ramos5@unmsm.edu.pe (J.A.R.-G.); renzo.vellasmin@edu.ufes.br (R.R.-V.); freddy.mendoza1@unmsm.edu.pe (F.M.-V.); 2 Departamento de Física, Universidade Federal do Espírito Santo, Vitória 29075-910, Brazil; passamaniec@yahoo.com.br; 3 Centro Brasileiro de Pesquisas Físicas (CBPF), R. Xavier Sigaud, 150, Urca, Rio de Janeiro 22290-180, Brazil; nomifsc@cbpf.br

**Keywords:** ^57^Fe Mössbauer spectrometry, magnetic nanoparticles, polymer functionalization, short-term acute assay

## Abstract

Using a modified co-precipitation method, 11(2) nm γ-Fe_2_O_3_ nanoparticles functionalized with PSSNa [Poly(sodium 4-styrenesulfonate)] saloplastic polymer were successfully synthesized, and their structural, vibrational, electronic, thermal, colloidal, hyperfine, and magnetic properties were systematically studied using various analytic techniques. The results showed that the functionalized γ-Fe_2_O_3_/PSSNa nanohybrid has physicochemical properties that allow it to be applied in the magnetic remediation process of water. Before being applied as a nanoadsorbent in real water treatment, a short-term acute assay was developed and standardized using a *Daphnia magna* biomarker. The ecotoxicological tests indicated that the different concentrations of the functionalized nanohybrid may affect the mortality of the *Daphnia magna* population during the first 24 h of exposure. A lethal concentration of 533(5) mg L^−1^ was found. At high concentrations, morphological changes were also seen in the body, heart, and antenna. Therefore, these results suggested the presence of alterations in normal growth and swimming skills. The main changes observed in the *D. magna* features were basically caused by the PSSNa polymer due to its highly stable colloidal properties (zeta potential > −30 mV) that permit a direct and constant interaction with the *Daphnia magna* neonates.

## 1. Introduction

The presence of toxic metals exceeding the permissible level in water is a hot topic in environmental sciences due to all the short- and long-term human health problems that these substances can cause to plants and living organisms [1]. For instance, arsenic (arsenite and arsenaite, and organic species) and lead (Pb) are two of the most harmful toxic metals. They are frequently found in ionic states in an aqueous media and are reported to cause intoxication of the central nervous system and male infertility, kidney failure, and cancer [2,3,4]. In this regard, novel metamaterials (nanoadsorbents) that are able to efficiently uptake the toxic metals in a short time-frame must be developed, studied, and tested in laboratories. In this sense, magnetic adsorption method has gained popularity in water remediation due to its excellent adsorption results and its easy removal from the effluent by an external magnetic field [5,6]. The synthesis of nanoabsorbents is often conducted following a co-precipitation chemical route, involving low budgets and huge amounts of materials obtained in short synthesis time (min) [5].

Among the adsorbents, magnetic composites, specifically those combined with polymers, are generally synthesized following a core@shell arrangement, where the material surface charge can be suitable for electrostatic attraction. In particular, these magnetic-polymer composites have revealed a considerable removal capacity for Cr(VI), Cu(II), Pb(II), and dyes in real short exposure times [7,8]. However, the use of magnetic@polymer nanocomposites for water remediation raises an additional concern, namely, its ecotoxicological impact, since, as an organic/inorganic external agent, the nanocomposites can affect the biocenosis of living organisms present in water, and consequently alter the ecological balance [9,10]. Thus, considering their promising adsorbent, colloidal, and magnetic properties, an ecotoxicity study must be developed first to understand if there is a negligible or negative impact on ecosystems caused by the magnetic remediation with any nanocomposite. It should be pointed out that each magnetic nanohybrid exhibits intrinsic physicochemical properties depending on their synthesis route, and the ecotoxicity test should be always performed before their technological applications.

Regarding the polymer, it is important to stress that poly(sodium 4-styrene sulfonate) (PSSNa) is a synthetic polymer categorized as a primary microplastic, intentionally manufactured for various applications, i.e., to prevent sexually transmitted diseases or for in vitro inhibition of Zika virus replication [11,12]. As a water-soluble anionic polyelectrolyte, it can be used in emulsion polymerization [13] and in the fabrication of advanced materials [14,15]. There is a poor literature reporting PSSNa ecotoxicity, hence its release into the environment contributes to an enhancement of microplastic pollution, now seen as a big problem that needs to be treated urgently. Therefore, to assess the potential environmental impact of PSSNa, it is essential to follow the recommended usage guidelines and updated regulatory tests [16,17]. In real effluents, one of the most often living organisms found is the *Daphnia magna* (*D. magna*). It is a species commonly used in toxicological studies due to its high sensitivity to external agents and the fact that it ideally represents zooplankton [18]. Based on this assumption, preliminary nanoecotoxicity properties of a magnetic@polymer nanocomposite must be evaluated to know its lethal concentration (*LC*_50_) that allows a controlled water magnetic treatment, i.e., controlling the reasonable increase in the adsorbent dose without creating a substantial impact on the effluent environment. Consequently, this procedure limits an excess of spreading nanohybrids into the aqueous environment, hence mitigating their toxicity effects in water bodies.

Thus, in this work, for the first time, the synthesis and physicochemical characterization of γ-Fe_2_O_3_-PSSNa nanohybrids are being reported. In addition, their ecotoxicity properties were evaluated for 24 h using *D. magna* biomarkers to establish the lethal concentration (24h-*LC*_50_) of interest for environmental aquatic release, i.e., to show the permissible concentration that the presented nanohybrid may have in real adsorption applications during short exposure periods.

## 2. Materials and Methods

### 2.1. Synthesis of γ-Fe_2_O_3_@PSSNa Core–Shell Arrangement

Iron(II) sulfate heptahydrate (FeSO_4_•7H_2_O), Iron(III) chloride anhydrous (FeCl_3_), and the polymer Poly(sodium 4-styrenesulfonate), linear formula (C_8_H_7_NaO_3_S)_n_, PSSNa, reactives were purchased from Sigma Aldrich and used without additional purification. The co-precipitation route was chosen for the synthesis of γ-Fe_2_O_3_ NPs and functionalization with PSSNa. A molar ratio of 0.5 between Fe^2+^ and Fe^3+^ salts were added in distilled water [19]. In total, 500 mg of PSSNa were used as stabilizing agent. The PSSNa powder was poured into a flat-bottom flask with 250 mL of distilled water, previously heated at about 80° C in the magnetic stirrer (the dispersion was stirred vigorously at 550 rpm for 30 min). The iron salts dispersion was subsequently added to the flask. The final dispersion composed of iron salts plus PSSNa turned to a reddish tone after adding the iron salts. Immediately after that, 25 mL of 28% NH_4_OH were dropped and kept under the same temperature and time, yielding a black color, indicating the presence of Fe_3_O_4_ NPs. Thereafter, the stirrer was turned off and left to 300 K. The suspended solid was magnetically separated and washed several times to remove supernatant residues. The sample was dried at 70 °C in an oven. After that, the sample was pulverized, and the color slightly changed to a reddish color, an effect often found in our synthesis, and that is characteristic of the Fe_3_O_4_ to γ-Fe_2_O_3_ transition due to a rich oxidant atmosphere.

### 2.2. Characterization

X-ray data were obtained from an RIGAKU Ultima IV diffractometer operating with a Cu tube at 40 kV and 30 mA. In addition, the X-ray data were collected with a CuK_α_ radiation (λ = 1.5418 Å) using the Bragg–Brentano configuration. Identification of the Bragg diffraction peak was carried out using the software Match! v3 [20]. The Rietveld refinement was performed by employing the FullProf Suite. The instrument resolution function (IRF) was taken from the Al_2_O_3_ diffraction pattern. The diffractogram matches with the PDF 9006316 available on the Match database, which corresponds to the γ-Fe_2_O_3_ phase, with a lattice parameter a = 8.30 Å (initial value for refinement) and is described by the spatial group Fd $\bar{3}$ m.

Average particle size, particle size distribution (PSD), and their morphological characteristics were conducted using a 200 kV JEOL 2100F imaging microscope instrument from Tokyo, Japan. The equipment was operated in both scanning and transmission modes, and also with a high resolution. The PSD curve was obtained using a total of 800 to 1000 particles from 30 to 35 pictures. A log-normal distribution was applied, based on Ref. [21], to interpret the histograms. The polydispersity values were determined by calculating the standard deviation of the log-normal distribution.

The Raman spectra were obtained with micro-Raman Renishaw in a Via Raman spectrometer. The measurements were performed in a wide range up to 3000 cm^−^^1^ using an objective × 50. Two lasers of 633 nm and 785 nm were employed due to the high fluorescence caused by the polymer PSS. The initial power of 7.17 mW (633 nm) was set for the γ-Fe_2_O_3_@PSSNa spectra collection, and subsequent measurements were performed at 0.05%, 0.1%, 0.5%, 1%, 5%, 10%, and 50% of the initial laser power. A total exposition time per sample of 20 s was considered, consisting in two accumulations of 10 s each. Transmission-mode Fourier Transform Infrared Spectroscopy (FTIR, Thermo Scientific Nicolet iS50, Waltham, MA, USA) analyses were performed on both samples, utilizing a 4 cm^−1^ resolution instrument, within the wavenumber of 4000 to 400 cm^−1^. The absorbance UV-Vis spectrum was obtained at 300 K in the range of 200–300 nm using an AVANTES spectrometer (Apeldoorn, The Netherlands). The integration time was 30 ms, and an average of 100 repetitions were performed to compile the spectrum. Subsequently, the acquired data were exported for processing using the AvaSoft8 software version. The TG measurements were performed using a Shimadzu equipment (Kyoto, Japan). The samples were subjected to heating in a synthetic air atmosphere with a flux rate of 50 mL min^−1^ at a rate of 10 °C/min, from 28 °C to 500 °C.

At 77 K, N_2_ adsorption/desorption isotherms were measured with a Tristar 3000 sorptometer from Micrometrics. A degasification process was conducted on the samples at 300 °C for a duration of 5 h beforehand. Using the BET and BJH models, the textural properties, including the specific surface area and pore size distribution, were ascertained. The volume of pores was evaluated at P/P_0_ = 0.98. Transmission ^57^Fe Mössbauer spectra were obtained in a Janis Corporation Inc. (San Francisco, CA, USA). He-closed cycle setup at 300 K and 15 K. The Mössbauer spectrometer and the sample holder were synchronized by being placed in an antivibration system and securely attached. ^57^Cobalt (Co) embedded in a Rhodium (Rh) matrix, with an activity of 25 milliCuries (mCi), was adapted in a drive that operates with a sinusoidal wave form. While the source always remains at 300 K, the absorber could be cooled down to 15 K (the lowest temperature of our Mössbauer setup). The powder absorbers were enclosed in nylon sample containers, with their thicknesses carefully selected to match 0.1 mg of ^57^Fe per cm^2^. The Mosswinn 4.0i program [22] was used to fit the ^57^Fe Mössbauer spectra. Magnetic properties were obtained in a Physical Property Measurement System, with a vibrating sample magnetometer option. Zero-field-cooling (ZFC) and field-cooling (FC) magnetic hysteresis loops (ZFC or FC *M*(*H*) loops) were obtained at 300 K and 5 K for a maximum scan field 7 T (i.e., ±7 T). The FC *M*(*H*) loop was conducted, cooling down the sample from 300 K to 5 K under an applied field of 1 T. The FC *M*(*H*) experiments were carried out to investigate an existence of the exchange bias effect.

### 2.3. D. magna Culture

The culture was maintained under favorable environmental conditions to favor the permanence of the asexual reproductive phase [23]. The temperature of the culture remains at (20 ± 2) °C, with a photoperiod ratio of 8 h of light and 16 h of darkness. The pH was kept at (7.5 ± 0.5). The individuals were fed daily, and the aquariums were cleaned periodically. In order to obtain embryos and maintain their culture, the medium was maintained in optimal conditions, with continual monitoring of physicochemical parameters and weekly replacement of the live water. To ensure enough space in the culture media for subsequent generations, the older organisms were removed, and the sediments were cleaned two to three times per week.

### 2.4. D. magna Exposure Protocol and LC_50_ Acute Toxicity Determination

On the day prior to the nanohybrid exposition, *D. magna* with embryos were chosen to guarantee that the organisms were younger than 24 h during the experiments. A total of thirty neonates were chosen for each concentration of the γ-Fe_2_O_3_@PSSNa nanohybrid. Prior to that, the γ-Fe_2_O_3_@PSSNa nanohybrid was stirred for 10 min in separate glass beakers containing 200 mL of the culture media. The following nine different concentrations were used for the ecotoxicological experiments: 25, 50, 100, 200, 400, 600, 800, 1000, and 1200 mg L^−1^. The neonates were exposed to a 24 h exposure period in the laboratory, following the conditions described in the previous section. The organisms that survived were moved to a culture medium that did not include any γ-Fe_2_O_3_@PSSNa. They were then kept for the next 13 days, receiving a daily feeding of 2 mL of microalgae *Chlorella vulgaris*. The *LC*_50_ values of the exposed *D. magna* to γ-Fe_2_O_3_@PSSNa were calculated using a sigmoidal nonlinear fit, as has been suggested with the experimental data behavior for the mortality (%) vs. logarithm of the concentration plot.

### 2.5. Morphological Evaluation in D. magna

The morphological alterations in the *D. magna* population exposed to γ-Fe_2_O_3_@PSSNa were studied using a Greetmed model DN117M optical microscope equipped with a camera and ScopeImage 9.0 software. Following a 13-day period of exposure, the dimensions of the eye, antenna, heart, body, and tail structures were evaluated for all tested concentrations in order to compare the findings with the corresponding negative controls [24]. Furthermore, the statistical analysis employed the Student’s *t*-test, and the outcomes were visually depicted using box plot diagrams. Significant results were statistically defined as having *p*-values less than 0.05, using SPSS statistical software v27, and with a confidence interval percentage equal to 95%. For this morphological analysis, the concentrations used for the γ-Fe_2_O_3_@PSSNa were gradually increased by the following: 50 mg L^−1^, 100 mg L^−1^, 200 mg L^−1^, 400 mg L^−1^, and 800 mg L^−1^. The number of individuals counted for each concentration were 6, 13, 16, 5, and 2 daphnids, respectively. For the negative control (N.C.), the number of individuals counted were 26.

## 3. Results and Discussion

### 3.1. Rietveld Refinement and TEM Analysis

**Figure 1:**
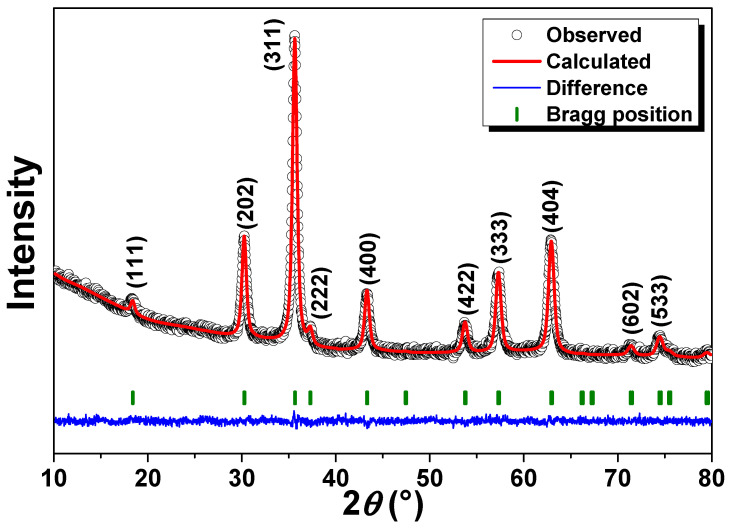
Refined X-ray diffractogram of the γ-Fe_2_O_3_@PSSNa nanohybrid. The Miller indexes of diffraction peaks are given in the top of each Bragg peak.

Figure 1 shows the Rietveld refined X-ray diffractogram, where the featured crystallographic planes (311), (111), (202), (400), (333), and (404) for the γ-Fe_2_O_3_ phase are clearly depicted. Table S1 resumes the refined parameters. The lattice parameter was found to be equal to a = 8.30 Å, a value that agrees with that reported in the literature for this phase [25]. The crystallite size has a value of 12 nm, indicating a nanometric regime of the γ-Fe_2_O_3_ phase. On the other hand, considering the polymer character of the PSSNa, no Bragg peak has been detected in X-ray experiments, i.e., this phase may behave amorphously from the X-ray point of view (absence of a long-range atomic order).

**Figure 2:**
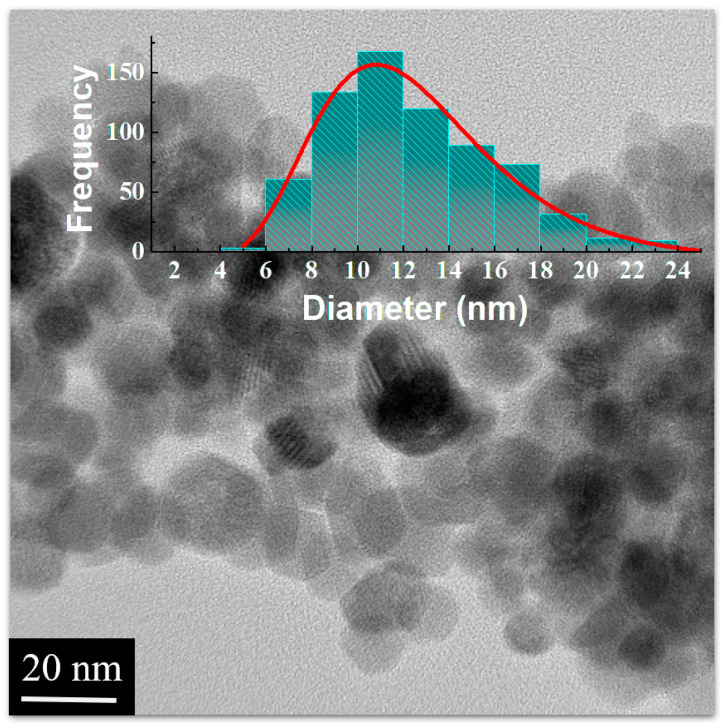
TEM image of the γ-Fe_2_O_3_@PSSNa nanohybrid. Inset shows the bar graph correlated to the particle size frequency.

Figure 2 displays the TEM image of the γ-Fe_2_O_3_@PSSNa nanohybrid, where the NPs depict their polydisperse behavior. The average particle has a value of 11(2) nm, which coincides with the estimated crystallite size from the X-ray data. The TEM image also suggests that PSSNa covers the γ-Fe_2_O_3_ NPs, and consequently it may be responsible for preventing γ-Fe_2_O_3_ agglomeration. This finding favors for a chemical stability of γ-Fe_2_O_3_ NPs as also reported in the literature [14,21].

### 3.2. Raman Analysis

**Figure 3:**
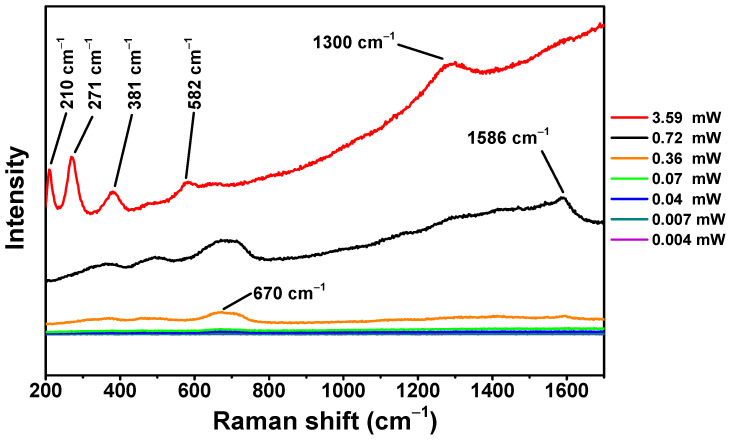
Raman spectra of the γ-Fe_2_O_3_@PSSNa nanohybrid measured at selected laser powers.

For the Raman analysis of the γ-Fe_2_O_3_@PSSNa nanohybrid, the optical range of 200 to 1700 cm^−1^ was chosen at several laser powers. In Figure 3, the spectrum corresponding to 3.59 mW (red line) presents two optical active modes at 210 cm^−1^ (A_1g_) and 271 cm^−1^ (E_g_) related to the polymorphic phase of low crystallized α-Fe_2_O_3_ [26]. The Raman modes at 381 and 582 cm^−1^ assign the contribution of the mixture of γ-Fe_2_O_3_ and low crystallized α-Fe_2_O_3_. The γ-Fe_2_O_3_ band can also be related to the additional peak found at 1300 cm^−1^, which appears close to that found by Hanesch at 1330 cm^−1^ [27]. In the literature, a threshold value for laser power has been reported depending on the γ-Fe_2_O_3_ magnetic structure and its combination with other nonmagnetic matrices [28,29,30]. Hence, we would like to point out that a bad interpretation of Raman spectra depends on this threshold value and must be individually determined for different magnetic nanohybrids, specifically for composites with γ-Fe_2_O_3_. More importantly, an intense laser power can result in the sample burning (carbon), hence not allowing us to correctly analyze the organic contribution.

In the signal that uses 0.72 mW (black line), the peak of 1586 cm^−1^ appears isolated, which could be attributed to the presence of the PSSNa polymer as previously reported at 1598 cm^−1^ [31]. The Raman modes located at 370, 460, 490, 670, and 1596 cm^−1^ were found for the 0.36 mW spectrum (orange line). The Raman mode that appears at 370 cm^−1^ is assigned to γ-Fe_2_O_3_ [26]. The contribution of the 460 and 490 cm^−1^ active modes produces a broad signal that can be attributed to γ-Fe_2_O_3_ [28,32]. The broad peak that appears between 670 cm^−1^ indicates the presence of γ-Fe_2_O_3_ [27]. Moreover, the isolated peak of 1596 cm^−1^ was attributed to PSSNa [27]. Below this laser power, it was not possible to identify the polymer contribution (this is explained due to the predominance of the iron-oxide phase in the sample). Hence, the 0.36 mW laser power value was stablished as the threshold laser power for this sample. Above this value, the polymorphic α-Fe_2_O_3_ phase has its onset ( γ→α transformation), and total crystallization can be achieved by tuning the laser power [23,28,29,30].

### 3.3. FTIR and Optical UV Vis Analysis

**Figure 4:**
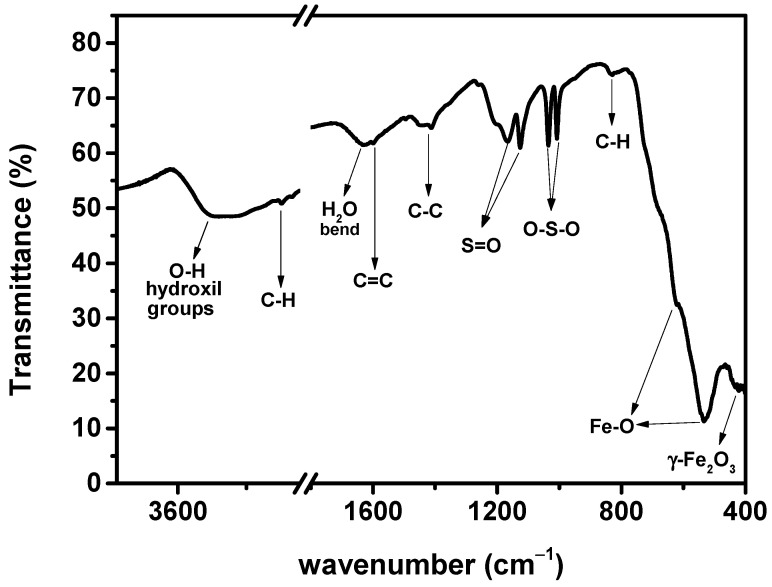
FTIR spectrum of the γ-Fe_2_O_3_@PSSNa nanohybrid recorded over the full range from 4000 to 400 cm^−1^.

The IR spectra of pure PSSNa and γ-Fe_2_O_3_@PSSNa are shown in Figure 4. IR peaks ca. 434, 534, and 619 cm^−1^ indicate the metal–oxygen bonding found in the tetrahedral sites, where the first peak could correspond to the only deformation of octahedral sites of the γ-Fe_2_O_3_ phase [33,34,35,36]. Bali et al. [34] have identified an IR peak at about 2925 cm^−1^ and associating it with the stretching vibration of the C-H bond. This IR peak value is close to that at 2920 cm^−1^ found in our IR spectrum of the γ-Fe_2_O_3_@PSSNa nanohybrid. In addition, the IR peak at 830 cm^−1^ corresponds to an out-of-plane vibration of the C-H groups in the benzene rings [37]. At about 3400 and 1630 cm^−1^, the broad IR peaks correspond to the stretching and bending vibrations for the O-H bond in the retained physiosorbed water [33,35].

The presence of benzene rings is expressed by the stretching of the C=C and C-C bonds at 1590 and 1430 cm^−1^, respectively. Funda et al. [38] associates these effects with the presence of PSSNa. The $-SO_{3}^{-}$ groups present in the PSSNa polymer appear around the peaks at 1124 and 1167 cm^−1^, while the typical symmetric stretching of O–S–O is located at 1006 and 1034 cm^−1^ [36,39]. This information confirms the presence of a PSSNa polymer functionalizing the γ-Fe_2_O_3_ phase.

Figure S1a shows the optical absorption spectrum of the γ-Fe_2_O_3_@PSSNa nanohybrid in the range of 250–550 nm. A pronounced decay and a concomitant rise in the optical spectrum at 350 nm may indicate the presence of a new peak at about 367 nm. Indeed, the presence of PSSNa occurs with a shift towards longer wavelengths in the range of 350 to 600 nm due to the presence of PSSNa aromatic groups by the π→π* transition [40]. Also, the position close to 370 nm exhibits the 6A1→4E transition for the γ-Fe_2_O_3_ phase [41], indicating a degeneracy in the energy region for these two phases of the sample (lack of energy resolution to solve these phases in the optical spectrum).

The forbidden band, *E_g_*, of the γ-Fe_2_O_3_@PSSNa nanohybrid, obtained with the UV-VIS absorption data, was experimentally determined from the absorption coefficient *α*, measured through the Tauc formula [42]:

(1) $$\left(\alpha h\nu \right)=A{\left(h\nu -E_{g}\right)}^{n}$$

where *A* is a constant, (*h* ν ) is the photon energy, and *n* can be either 1/2 for direct transmission or 2 for indirect transmission.

Figure S1b belongs to the indirect transmission graph with *n* = 1/2. This quantity was obtained by extrapolating a straight line over a straight portion of the curve, chosen between the range of 3.44 and 3.52 eV. The intersection of this straight line with the *x*-axis gives a value of *E_g_* = 1.42 eV. It is less than the 2.0 eV reported for bulk γ-Fe_2_O_3_ [43]. Consequently, this reduction could be attributed to size-finite effects that directly influence the *E_g_* value, as reported in the literature [43].

### 3.4. Colloidal, Thermal, and Textural Properties

PSSNa is commonly used to improve the dispersion stability of ceramic slurry, such as dispersants of alumina, titanium dioxide, zirconia, and other metal-oxide ceramics [44]. Regarding the value of the hydrodynamic diameter of PSSNa, different values have been reported. For a PLL/PSSNa complex, a value of 2.1 μm at 300 K and pH = 5 was reported [45], while a hydrodynamic radius of a bare molecule equal to 2.5 nm is found in Ref. [46]. In this study, the commercial polymer PSSNa has a hydrodynamic diameter value of 5.2 μm, which is twice the value found in Ref. [45]. On the other hand, the γ-Fe_2_O_3_@PSSNa nanohybrid shows a value of 210 nm (or 0.21 μm), which is ten times smaller than 2.1 μm found for the PLL/PSSNa complex. Also, it is important to mention that no substantial agglomeration of the functionalized γ-Fe_2_O_3_ has been occurred after a long time [47], suggesting that the polymer plays an important role on the NP agglomeration process.

**Figure 5:**
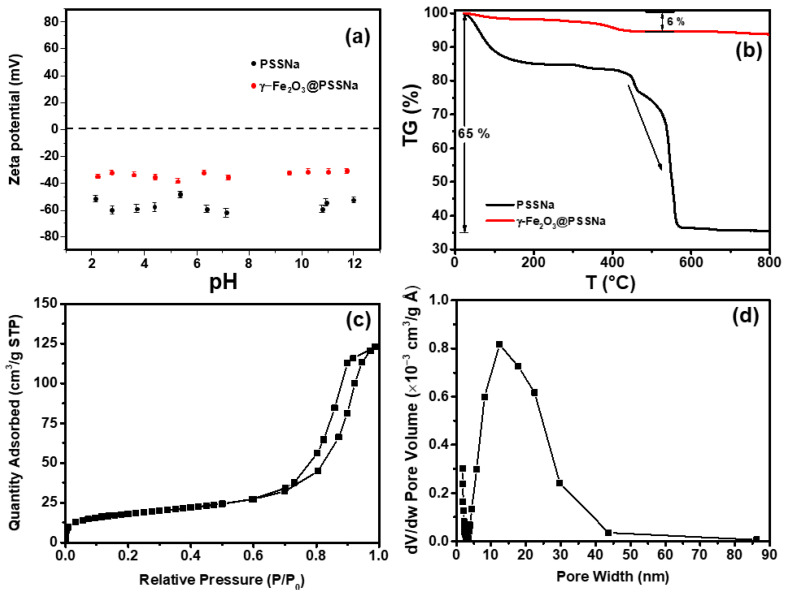
(**a**) Zeta potential and hydrodynamic size distribution of the PSSNa and γ-Fe_2_O_3_@PSSNa samples. Error < 5%. (**b**) TG measurement for the PSSNa and γ-Fe_2_O_3_@PSSNa samples. The arrow indicates the abrupt weight-loss decay after 400 °C. (**c**) N_2_ adsorption–desorption isotherm for the γ-Fe_2_O_3_@PSSNa nanohybrid, and (**d**) pore size distribution for the γ-Fe_2_O_3_@PSSNa nanohybrid.

Colloidal stability was evaluated by analyzing the Zeta potential vs. pH plot, shown in Figure 5a. For the bare PSSNa saloplastic, a negative charge was manifested in the entire pH range, with high stability values varying between −40 and −70 mV. Similar negative values of −41 to −52 mV have been reported for the PSSNa/MWNT in the KCl solution, which has a pH close to neutral [48]. Furthermore, a high Zeta potential for the PSSNa-coated Au of (−47.9 ± 3.2) mV was reported [49]. Thus, regarding the γ-Fe_2_O_3_@PSSNa nanohybrid, a negative Zeta potential was detected throughout the measured pH range, with values ranging between −30 and −40 mV. This phenomenon is mainly attributed to the effect of PSSNa on colloidal stability, since the γ-Fe_2_O_3_ NPs generally have isoelectric points greater than 6. For example, a p.z.c. value of 6.3 for the 12 nm MZ0 composite [5] and 8.3 for the 6 nm γ-Fe_2_O_3_ NPs have been reported [50]. These results suggest that both γ-Fe_2_O_3_-based NP materials demonstrated a remarkable colloidal stability with a marked predominance of negative surface potential, similar to the results obtained from the 12 nm γ-Fe_2_O_3_@PSSNa nanohybrid, here studied.

Thermogravimetric measurements for the PSSNa and γ-Fe_2_O_3_@PSSNa samples are shown in Figure 5b. For pure PSSNa, a total weight loss of 65% was observed. Sulfonic-acid group (SO_3_H) desulfonation and polystyrene degradation in the 200–550 °C range were related to the abrupt decrease after 400 °C. Up to roughly 800 °C, the primary carbon-chain skeleton was observed [51]. For the γ-Fe_2_O_3_@PSSNa nanohybrid, the quantified weight loss of 6% has the same change as that of pure PSSNa, and the small loss is because the PSSNa makes up a small percentage of the mass of γ-Fe_2_O_3_@PSSNa.

Figure 5c shows the hysteresis adsorption–desorption isotherm obtained at 77 K for the γ-Fe_2_O_3_@PSSNa nanohybrid. The found textural properties were BET area of 66 m^2^/g, pore volume of 0.186 cm^3^/g, and pore diameter equal to 20 nm. The pore distribution, which was estimated using the BJH approach, showed a broad range of pores, as displayed in Figure 5d. The isotherm curve exhibited the distinctive shape of an IV-type isotherm, which is associated with mesoporous materials [52].

### 3.5. Magnetic Studies

**Figure 6:**
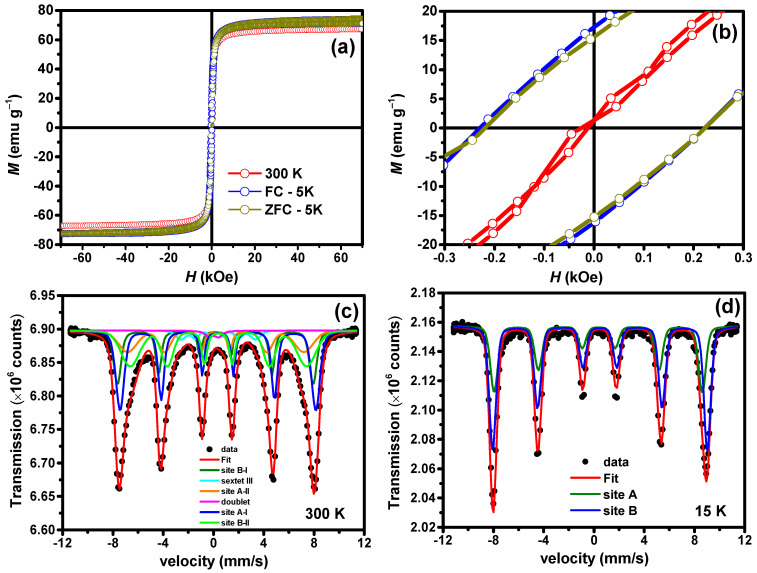
(**a**) FC and ZFC *M*(*H*) curves for the γ-Fe_2_O_3_@PSSNa nanohybrid recorded at 300 K and 5 K. (**b**) Zoomed region for the *M*(*H*) curves displayed in (**a**). (**c**) ^57^Fe Mössbauer spectra at 300 K and (**d**) 15 K for the γ-Fe_2_O_3_@PSSNa nanohybrid.

Figure 6a shows the ZFC and FC *M*(*H*) curve recorded at 300 and 5 K for the γ-Fe_2_O_3_@PSSNa nanohybrid. The zoomed region in Figure 6b shows the magnetic behavior of γ-Fe_2_O_3_@PSSNa at 5 K. 300 K *M*(*H*) curve shows a low coercive field (*H*_C_) of 100 Oe; see also Table S2. It can be explained assuming that the distribution of magnetic particles is weakly interacting and/or a result of ferrimagnetic-like character of the of γ-Fe_2_O_3_ NPs. The Law of Approach to Saturation (LAS), given by Equation (2) [24], was used to fit the *M*(*H*) curves in the range of +20 to +70 kOe:

(2) $$M=M_{S}\left(1-\frac{b}{H^{2}}\right)+\chi H.$$

where *Ms* is the saturation magnetization, χ is the high-field susceptibility, and the effective anisotropy constant, $K_{eff}$ , can be obtained by replacing the value of *Ms* in $b=\frac{4}{15}\left(\frac{K_{eff}^{2}}{M_{S}^{2}}\right)$ .

Table S2 summarizes the magnetic parameters obtained with the LAS. In the ZFC protocol, a *H*_c_ field of 0.22 kOe and a remnant magnetization *M_r_* of =15.45 emu g^−1^ were calculated. The fitting of FC *M*(*H*) data recorded at 5 K displays similar values, i.e., *H_c_* = 0.22 kOe and *M_r_* = 16.73 emu g^−1^, indicating the absence of magnetic interactions that could result in the exchange of bias effect sometimes found in an ensemble of nanoparticles (it should favor a shift of the *M*(*H*) loop towards the negative field axis [19]). The *Ms* showed value of 66.0(1) emu g^−1^ for 300 K and 71.0(1) emu g^−1^ at 5 K for both ZFC and FC *M*(*H*) loops. The obtained *Ms* values agree with bulk γ-Fe_2_O_3_ [53]. The values for *χ*, obtained from the fittings and shown in Table S2, indicate that the saturation regime was roughly reached, and the $K_{eff}$ values are like those reported in the literature for nanometric Fe-oxide based samples [19,24].

Figure 6c,d presents the 300 K and 15 K ^57^Fe Mössbauer spectra of the γ-Fe_2_O_3_@PSSNa nanohybrid. Table S3 reports the fitted hyperfine parameters. The 300 K spectrum was fitted with six components. Four sextets were used to represent the distribution of particle sizes of the γ-Fe_2_O_3_ NPs, also seen in TEM images. Indeed, the bulk cubic spinel γ-Fe_2_O_3_ phase only shows two well-defined sites: tetrahedral (A) and octahedral (B) sites of the ion Fe^3+^ spins. However, considering the large distribution particle sizes of the γ-Fe_2_O_3_ NPs, part of these NPs (<10 nm) starts to enter in a spin relaxation regime at 300 K, broadening the absorption line. Thus, two additional sextets were added to represent this fraction of NPs. The five (doublet) and six (III) subspectra, added to fit the 300 K spectrum, can, respectively, be associated with very small γ-Fe_2_O_3_ NPs in the superamagnetic regime and uncompensated surface Fe^3+^, as proposed by Zakharova et al. [54]. To estimate the thickness of this shell, spherical particles were considered, such as the surface to volume ratio, which can be assumed to be $V_{e}/V=3\Delta r/r$ , where $V_{e}=4\pi r^{2}\Delta r$ and $V=4/3\pi r^{3}$ . Thus, the thickness of Fe^3+^ shell spins, defined by Equation (3) [54], can be estimated:

(3) $$\Delta r=\frac{total\;area\;fraction}{6}\times \langle D_{TEM}\rangle$$

The total area fraction is indicated in Table S3, i.e., the contribution of the surface site area A to B was added, giving a total of 43% and $\langle D_{TEM}\rangle =11.4\;nm$ , resulting in an average thickness of Δr=0.91 nm .

The 15 K spectrum in Figure 6d only displays the two static magnetic sextets that agree with a highly stoichiometric γ-Fe_2_O_3_, with remarkable asymmetry between lines 1:6. As expected, in pure samples, the values for CS and *B_hf_* are close to each other. In addition, the relative fraction (R.A.A.) for the A and B sites (37% and 63%, respectively) are close to the stoichiometric formula (1.6) [50].

### 3.6. Ecotoxicological Analysis in D. magna

#### 3.6.1. *LC*_50_ of γ-Fe_2_O_3_@PSSNa Nanohybrid

**Figure 7:**
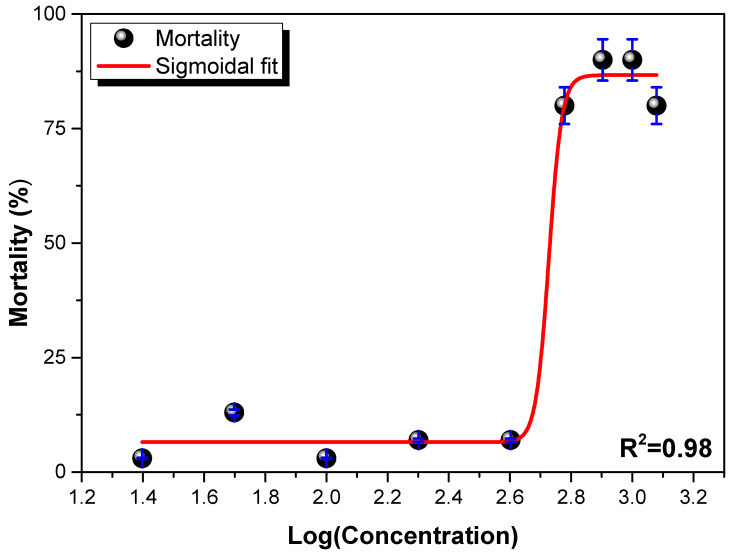
Sigmoidal fit for the mortality of *D. magna* exposed to the γ-Fe_2_O_3_@PSSNa nanohybrid.

The *LC*_50_ value found using the sigmoidal non-linear fit was 533(5) mg L^−1^, as seen in Figure 7. This value is close to 550 mg L^−1^ found for the ternary nanohybrid reported in the literature [24]. Table 1 presents a brief data collection of the parent systems used for comparison of the ecotoxicological profile.

In short, the ecotoxicological impact will depend on NPs size, as shown in Table 1. On the other hand, it should be said that the colloidal stability and hydrodynamic diameter are parameters not often reported in various studies with NPs. However, in the present study, the γ-Fe_2_O_3_@PSSNa nanohybrid depicted zero p.z.c. and highly stable Zeta potential values. These results indicate that the nanohybrid will interact for more time with the neonates, affecting their mortality. Indeed, γ-Fe_2_O_3_ (bulk or nano) by itself depicts low colloidal stability [23], tending to quick sedimentation, therefore, its colloidal stability will certainly affect the *LC*_50_ value, as demonstrated in this work.

**Figure 8:**
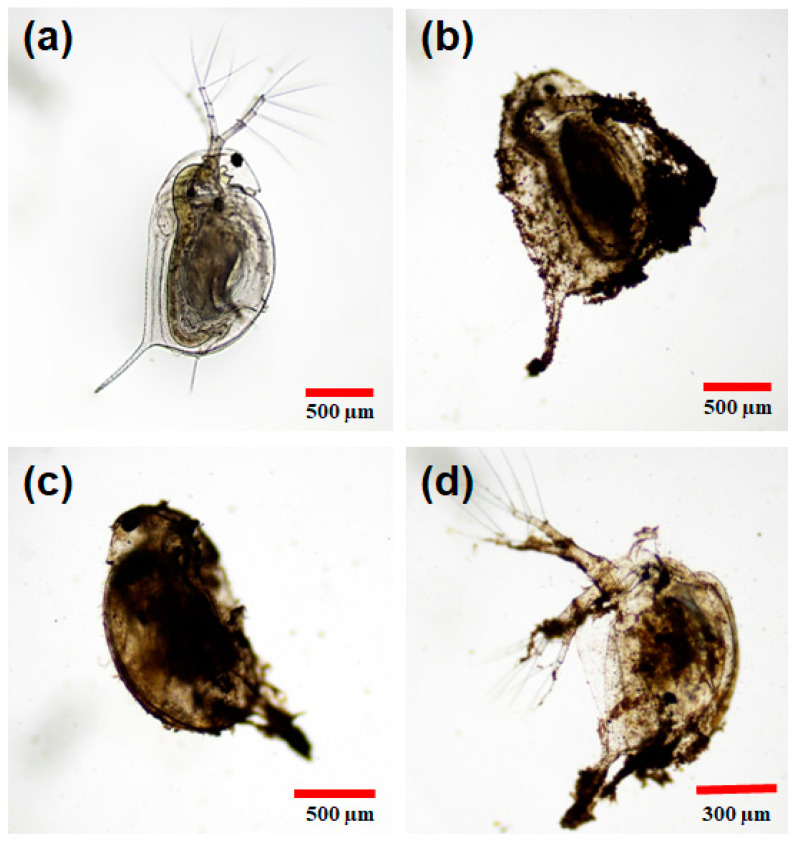
Optical Images of the direct interaction of γ-Fe_2_O_3_@PSSNa on *D. magna*. (**a**) An individual of the N.C. without exposure to γ-Fe_2_O_3_@PSSNa. In (**b**,**c**), two dead individuals exposed to γ-Fe_2_O_3_@PSSNa at 800 and 600 mgL^−1^, respectively. In (**d**), a molt is observed (only exoskeleton), evidencing the affinity of γ-Fe_2_O_3_@PSSNa to the chitinous exoskeleton; the applied concentration was 400 mg L^−1^. Photographs were taken with the 4× objective under a conventional bright-field optical microscope.

Figure 8 shows the visual effects of the γ-Fe_2_O_3_@PSSNa nanohybrid after 24 h of exposure. The N.C., without exposure to γ-Fe_2_O_3_@PSSNa, is shown in Figure 8a. It can be noted that the limbs are free and there is no obstruction in the digestive tract. This observation differs from what is shown in the following images, where it is seen that the NPs obstruct the limbs and adhere to the chitinous exoskeleton blocking the ability to move. Figure 8b displays the exoskeleton with the NPs with a concentration of γ-Fe_2_O_3_@PSSNa of 800 mg L^−1^. Figure 8c depicts *D. magna* immersed in a medium with a concentration of 600 mg L^−1^, whereas Figure 8d presents the residues left in the exoskeleton at a concentration of γ-Fe_2_O_3_@PSSNa of 400 mg L^−1^.

#### 3.6.2. Morphological Analysis in *D. magna*

**Figure 9:**
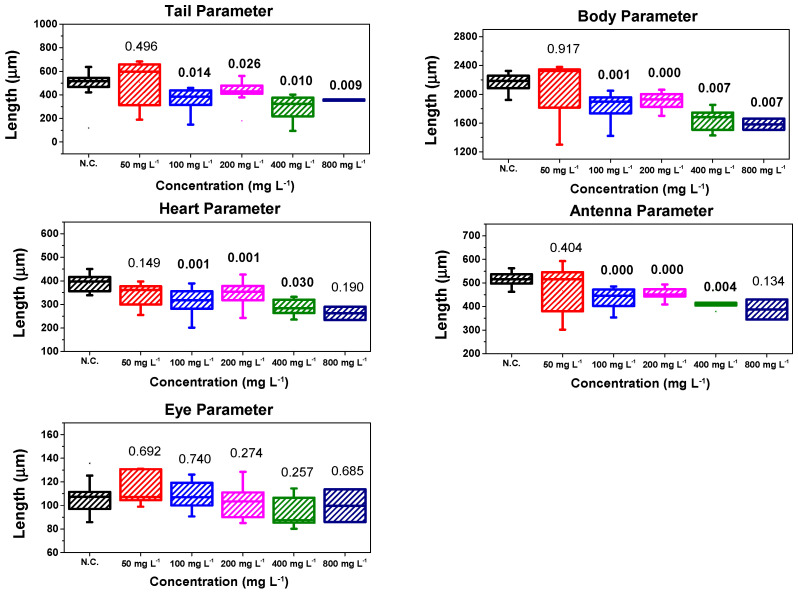
Box plots for the morphological parameters after exposing *D. magna* to γ-Fe_2_O_3_@PSSNa. N.C. stands for negative control.

After the control period of the surviving *D. magna*, morphological analysis was performed. Once it was confirmed that the morphological parameter data followed a normal distribution, Student’s *t*-test was applied. The results of the morphological analysis are shown in a box plot, as in previous ecotoxicity research [23,24,58,63]. Figure 9 shows the results of morphological analysis of the γ-Fe_2_O_3_@PSSNa nanohybrid for the concentrations of 50 mg L^−1^, 100 mg L^−1^, 200 mg L^−1^, 400 mg L^−1^, and 800 mg L^−1^, after the established control time, which was 13-days after exposure of *D. magna* to γ-Fe_2_O_3_@PSSNa.

The results suggest that only the concentration of 50 mg L^−1^ yields no significant change for any of their morphological parameters, while the eye parameter has no significant change regardless of the concentrations applied. Thus, the latter indicates that *D. magna* can maintain orientation after exposure to γ-Fe_2_O_3_@PSSNa.

On the other hand, the morphological parameters (heart, tail, body, and antenna) showed a significant change with respect to the concentrations, except for the 800 mg L^−1^ concentration, in which the antenna and heart parameters did not present a substantial modification. It is important to mention that these changes in morphological parameters may be related to genotoxicological damage occurring in *D. magna*, as also observed in *Chironomus riparius* using graphene oxide, in which there was an alteration in genes involved in the metabolism [64]. Thus, it can be inferred that it probably occurred after *D. magna* ingested γ-Fe_2_O_3_@PSSNa, which has an intermediate size between 20 nm and 70 μm, as suggested by Matson [62]. This effect would also explain why *D. magna* was reduced in size at concentrations of 100 mg L^−1^, 200 mg L^−1^, 400 mg L^−1^, and 800 mg L^−1^ of γ-Fe_2_O_3_@PSSNa with respect to the negative control.

**Figure 10:**
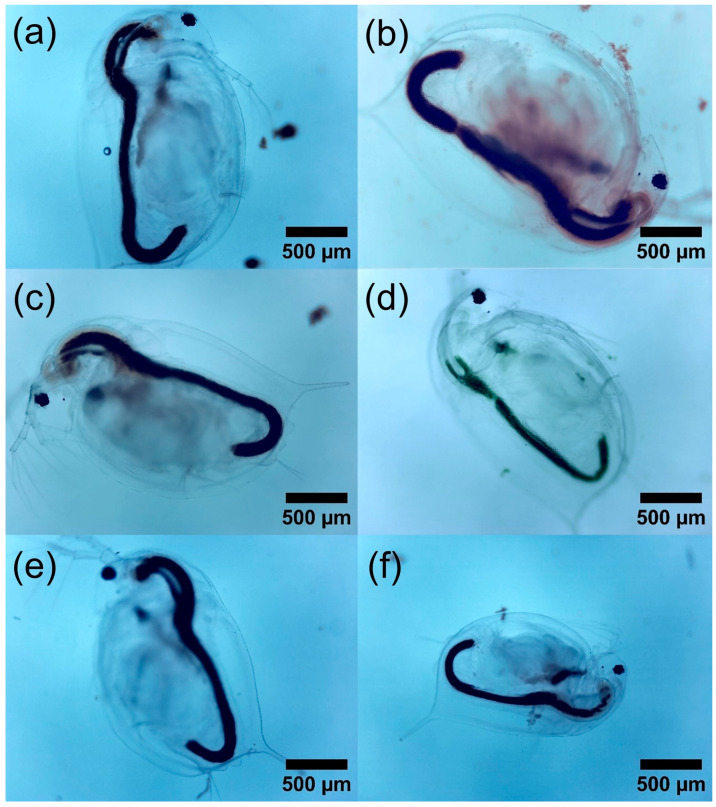
*D. magna* after 13-days of exposure to (**a**) N.C., (**b**) 50 mg L^−1^, (**c**) 100 mg L^−1^, (**d**) 200 mg L^−1^, (**e**) 400 mg L^−1^, and (**f**) 800 mg L^−1^.

Therefore, it can be said that the PSSNa polymer is likely the main cause of a significant change in the other morphological parameters, if one considers the results reported for γ-Fe_2_O_3_ NPs functionalizing graphene oxide that only show significant changes at high γ-Fe_2_O_3_ concentrations [58]. These results in the morphological parameters of *D. magna* are interesting, since these significant changes in the morphological parameters of the tail and antenna would affect the swimming of *D. magna*, while the reduced size of the heart may be due to the acute exposure to γ-Fe_2_O_3_@PSSNa, as shown in previous research that reported that anomalies in embryonic development can affect the morphology of *D. magna* [65]. Moreover, these changes are more sensitive in embryos than in juveniles of *D. magna* [66]. Although this study used neonates and not embryos or young individuals of *D. magna*, it is possible that the exposure of *D. magna* to γ-Fe_2_O_3_@PSSNa caused these significant changes in their morphological parameters, as it developed in the days following γ-Fe_2_O_3_@PSSNa exposure, whose significant *p*-values are shown in the box plots in Figure 9. Finally, a qualitative comparison is made using the images of *D. magna* on the last-control day, as shown in Figure 10a–f.

## 4. Conclusions

γ-Fe_2_O_3_ NPs functionalized with PSSNa polymer were successfully synthesized using a modified co-precipitation route. Different experimental methods were initially applied to characterize their structural, vibrational, electronic, thermal, colloidal, hyperfine, and magnetic properties. Magnetization and ^57^Fe Mössbauer data suggested that the γ-Fe_2_O_3_@PSSNa composite shows ferrimagnetic-like features that allow for magnetic remediation. Infrared and Raman indicated that the polymer covered the γ-Fe_2_O_3_ NPs, yielding chemical stability and reducing agglomeration. This material was tested for its ecotoxicological impact on *D. magna*. The results of the morphological analysis revealed that the γ-Fe_2_O_3_@PSSNa nanohybrid, although not totally toxic since its 24 h *LC*_50_ is 533(5) mg L^−1^, undergoes significant changes in the morphological parameters of the *D. magna* at concentrations below 800 mg L^−1^ after 13-days of exposure. It is relevant to note that, at all concentrations examined, no noticeable change in the ocular parameter was evident. Furthermore, at a concentration of 50 mg L^−1^, no relevant alterations were observed in any of the morphological parameters here evaluated. These findings indicate the optimal concentration of the γ-Fe_2_O_3_@PSSNa composite for the magnetic remediation of contaminated water, i.e., a permissible value that can be applied with a big impact on living organisms found in ordinary effluents.

## Figures and Tables

**Table 1:** *LC*_50_ values for similar nanosystems and polymers to the γ-Fe_2_O_3_@PSSNa nanohybrid exposed to *D. magna*. (n.d. = not determined). * SAMN: Surface Active Maghemite Nanoparticle.

Parent System	Mean Particle Size in Aqueous Media (nm)	NPs Source	Exposition Time (h)	*LC*_50_ (mg L^−1^)	Reference
Fe_3_O_4_ NPs (6 nm)	n.d.	Synthesized	48	2.3	[55]
SAMN * (11 (2) nm)	5–20	Synthesized	48	1.25–40	[56]
PVP-IONP (6.1 (6) nm)	82.3	Synthesized	48	9750	[57]
MWCNTs-γ-Fe_2_O_3_ (13.8 (6) nm for γ-Fe_2_O_3_)	n.d.	Synthesized	24	381.8	[58]
GO-γ-Fe_2_O_3_ (10.4 (2) nm for γ-Fe_2_O_3_)	n.d.	Synthesized	24	0.9	[58]
Fe_2_O_3_ (20–40 nm)	n.d.	Commercial	96	163.21	[59]
NP-Fe_3_O_4_ (<20 nm)	n.d.	Synthesized	48	977.24	[60]
PS (<75 μm)	n.d.	Commercial	48	78.94	[61]
PAO_2_N (52 nm)	56	Commercial	<24	<75	[62]
γ-Fe_2_O_3_@PSSNa (11(2) nm)	232.2	Synthesized	24	533(5)	This work

## Data Availability

The original data of this research can be requested any time to the corresponding author’s email address: emanriquec@unmsm.edu.pe.

## Supplementary Materials

The following supporting information can be downloaded at: https://www.mdpi.com/2073-4360/16/11/1581/s1, Table S1: Rietveld refined parameters for the γ-Fe_2_O_3_@PSSNa nanohybrid. Refinement and statistical parameters, R_p_(%) as profile refinement, R_wp_ (%) as weighted profile residual, R_exp_(%) as the expected profile residual, and goodness of the fit, $\chi^{2}$ . B is the temperature factor and Occ. represents the fraction occupancies. Table S2. Magnetic parameters obtained from LAS equation. n.d. not defined. Table S3. Hyperfine parameters obtained from the fits of the 300 K and 15 K ^57^Fe Mössbauer spectra of the γ-Fe_2_O_3_@PSSNa nanohybrid. R.A.A. is the relative absorption area, B_hf_ the hyperfine magnetic field, CS (mm/s) the center shifts respective vs. Fe at 300 K (0.114 mm/s), Q (mm/s) is the quadrupolar shifting, W is the Lorentzian line width, and σ the width of Gaussian distribution. Figure S1: (a) Absorbance UV Vis spectrum, and (b) forbidden band determination from the intersection of photon energy (eV).
