# Supplementary material for: Synthesis and Characterization of Maghemite Nanoparticles Functionalized with Poly(Sodium 4-Styrene Sulfonate) Saloplastic and Its Acute Ecotoxicological Impact on the Cladoceran Daphnia magna
Source: Polymers (Basel). 2024 Jun 3;16(11):1581. doi: 10.3390/polym16111581 (PMC11174764; doi:10.3390/polym16111581)
Supplement: Supplementary file 1 [file polymers-16-01581-s001.zip › polymers-2987434-supplementary.pdf]

polymers

Article

Synthesis and Characterization of Maghemite Nanoparticles Functionalized with Poly(Sodium 4-Styrene Sulfonate) Saloplastic and Its Acute Ecotoxicological Impact on the Cladoceran *Daphnia magna*

Juan A. Ramos-Guivar [1], Renzo Rueda-Vellasmin [1,2], Erich V. Manrique-Castillo [1,2,*], F. Mendoza-Villa [1], Noemi-Raquel Checca-Huaman [3] and Edson C. Passamani [2]

[1] Grupo de Investigación de Nanotecnología Aplicada para Biorremediación Ambiental, Energía, Biomedicina y Agricultura (NANOTECH), Facultad de Ciencias Físicas, Universidad Nacional Mayor de San Marcos, Av. Venezuela Cdra 34 S/N, Ciudad Universitaria, Lima 15081, Peru; juan.ramos5@unmsm.edu.pe (J.A.R.-G.); renzo.vellasmin@edu.ufes.br (R.R.-V.); freddy.mendoza1@unmsm.edu.pe (F.M.-V.)
[2] Departamento de Física, Universidade Federal do Espírito Santo, Vitória 29075-910, Brazil; passamaniec@yahoo.com.br
[3] Centro Brasileiro de Pesquisas Físicas (CBPF), R. Xavier Sigaud, 150, Urca, Rio de Janeiro 22290-180, Brazil; nomifsc@cbpf.br
* Correspondence: emanriquec@unmsm.edu.pe

Supplementary Tables

Table S1. Rietveld refined parameters for the γ-Fe_2O_3@PSSNa nanohybrid. Refinement and statistical parameters, R_p(%) as profile refinement, R_{wp} (%) as weighted profile residual, R_{exp}(%) as the expected profile residual, and goodness of the fit, χ^2. B is the temperature factor and Occ. represents the fraction occupancies.

Sample		Wyckoff positions			Temperature and occupancy factors		Crystallite size (nm)	Statistical parameters
		x	y	z	B	Occ.		
γ-Fe_2O_3 + PSSNa	Fe-tet	0.1250	0.1250	0.1250	0.9887	0.0447	12	R_p= 189 %
	Fe-Oct	0.5000	0.5000	0.5000	0	0.0739		R_{wp}= 69 %
	O	0.25	0.25	0.25	0	0.1449		R_{exp}= 66 %
								χ^2 =1.1

Table S2. Magnetic parameters obtained from the LAS equation. n.d. equal to not defined.

T (K)	M_r (emu g^{-1})	H_c (kOe)	M_s (emu g^{-1})	K_{eff} × 10^5 (J m^{-3})	χ (emu g^{-1} kOe)	R^2
300	n.d.	0.10	66.0(1)	0.78	0.02(4)	0.999
5 ZFC	15.45	0.22	71.0(1)	1.08	0.00(4)	0.999
5 FC	16.73	0.22	71.0(1)	1.08	0.00(4)	0.998

Table S3. Hyperfine parameters obtained from the fits of the 300 K and 15 K ^{57}Fe Mössbauer spectra of the γ-Fe_2O_3@PSSNa nanohybrid. R.A.A. is the relative absorption area, B_{hf} the hyperfine magnetic field, CS (mm/s) the center shifts respective vs. Fe at 300 K (0.114 mm/s), Q (mm/s) is the quadrupolar shifting, W is the Lorentzian line width, and σ the width of Gaussian distribution.

	R.A.A. (%)	CS (vsFe)	Bhf (T)	σ (T)	Q (mm/s)	W (mm/s)
300 K						

I,A	19(2)	0.26(1)	48.4(1)	1.2(1)	0.00(1)	0.37(1)
I,B	31(2)	0.47(1)	48.4(1)	1.5(1)	0.00(1)	0.37(1)
II,A	17(2)	0.30(1)	43.6(1)	4.1(2)	0.00(1)	0.37(1)
II,B	26(2)	0.48(1)	43.6(1)	4.1(2)	0.00(1)	0.37(1)
III	4(2)	0.44(4)	18.5(3)	2.0(2)	-0.17(5)	0.35(1)
doublet	3(2)	0.15(4)	-	-	0.8(1)	0.70(1)
15 K						
A	37(2)	0.36(5)	51.5(2)	1.1(1)	0.00(2)	0.24(1)
B	63(2)	0.48(5)	53.1(2)	1.1(1)	0.00(2)	0.24(1)

Supplementary Figures

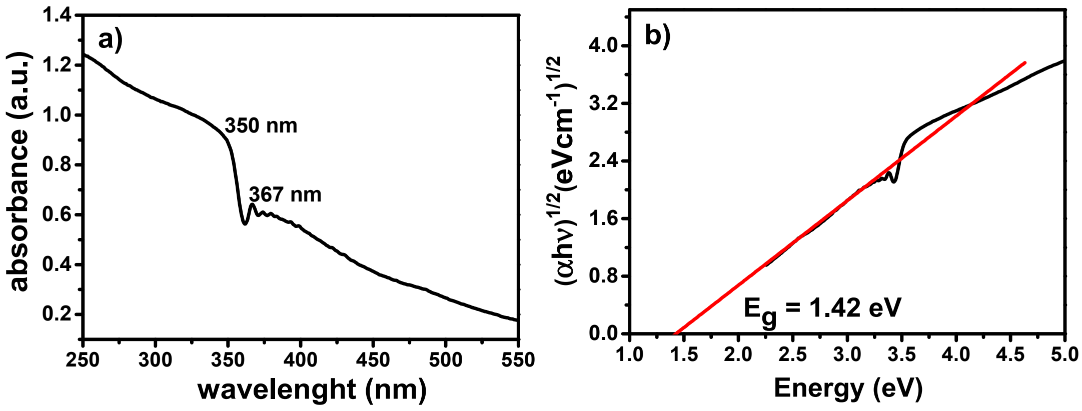

Figure S1. a) Absorbance UV Vis spectrum, and b) forbidden band determination from the intersection of photon energy (eV).
